# Supplementary material for: Content-rich biological network constructed by mining PubMed abstracts
Source: BMC Bioinformatics. 2004 Oct 8;5:147. doi: 10.1186/1471-2105-5-147 (PMC528731; doi:10.1186/1471-2105-5-147)
Supplement: Additional File 2 — The original results of the above study (non-essential files are deleted to keep the file size under the limit set by BMC bioinformatics). [file 1471-2105-5-147-S2.bz2 › chilibotAdditionalFile2/dip10/113ID8645696E457/html/ATPA_ATPH.html]

 


 **ATPA** and **ATPH** 
  
Found 15 abstracts in PubMed, retrieved 10.  
 

 What does Google say? 
 PDF only 
| .edu only 

---

**Interactive relationship** (e.g. stimulation, inhibition, etc)

**Stimulatory relationship**- However, there is no fixed stoichiometry of coupling mutations in  **atpH**  lead to increases in the translation ratio  **atpA**   **atpH**  of up to approximately 40 fold.  Ref: 7517937 J Biol Chem, 1994
**Neutral relationship**- 8 762 4766 indicate that primarily ribosomes reinitiating after termination on  **atpH**  are responsible for inducing refolding of the  **atpA**  TIR.  Ref: 7517937 J Biol Chem, 1994
- The gene pair atpHA is tightly coupled, whereby  **atpA**  is translated at least three times more efficiently than  **atpH** .  Ref: 7517937 J Biol Chem, 1994
- This paper describes the  **atpA**  gene cluster in the C. reinhardtii chloroplast genome, which includes the  **atpA** , psbI, cemA, and  **atpH**  genes, encoding the alpha subunit of the coupling factor 1 CF1 ATP synthase, a small photosystem II polypeptide, a chloroplast envelope membrane protein, and subunit III  [ **ATPH** ]  of the CF0 ATP synthase, respectively.  Ref: 9625716 Plant Physiol, 1998

**Non-interactive relationship** (e.g. studied together, co-existance, homology, etc.)

- Five genes, atpE, atpF,  **atpH** ,  **atpA**  and atpG, were shown to be translationally coupled to various degrees of tightness.  Ref: 1830358 Mol Microbiol, 1991
- These fragments contain six closely linked reading frames that were identified as atpI,  **atpH** , atpG, atpF, atpD, and  **atpA** , coding for subunits IV, III, II, I, delta, and alpha, respectively.  Ref: 1532839 J Mol Biol, 1992
- We show that promoters precede the  **atpA** , psbI, and  **atpH**  genes, but not the cemA gene,.  Ref: 9625716 Plant Physiol, 1998
- Individual subunits of ATP synthase, encoded by the eight genes of the atp operon  **atpA**  through  **atpH** , have been found to be synthesized at a 10 fold range in molar amounts D.L.  Ref: 7690747 J Bacteriol, 1993
- No genes for photosynthetic functions have been found, except for sequences encoding six subunits of the ATP synthase  **atpA** , atpB, atpE, atpF,  **atpH** , and atpI.  Ref: 12111556 Mol Genet Genomics, 2002
- The C. thermoaceticum atp operon contained nine genes arranged in the order atpI i, atpB a, atpE c, atpF b,  **atpH**  delta,  **atpA**  alpha, atpG gamma, atpD beta, and atpC epsilon.  Ref: 9171425 J Bacteriol, 1997
- Eight genes atpI, atpB, atpE 1, atpE 2, atpE 3, atpF,  **atpH** , and  **atpA**  upstream of and contiguous with the previously described genes atpG, atpD, and atpC were cloned from chromosomal DNA of Acetobacterium woodii.  Ref: 10567365 J Biol Chem, 1999
- The linear order of these genes, 5 rps2 atpI  **atpH**  atpF  **atpA**  rps18 3, encoding ribosomal protein S2, chloroplast ATP synthase subunits CF0IV, CF0III, CF0I, CF1 alpha and ribosomal protein S18, respectively, is similar to the equivalent operons of prokaryotes, cyanelles and land plant chloroplasts.  Ref: 8435857 Curr Genet, 1993
